# Human-Animal Interaction Research: Progress and Possibilities

**DOI:** 10.3389/fpsyg.2019.02803

**Published:** 2019-12-20

**Authors:** James A. Griffin, Karyl Hurley, Sandra McCune

**Affiliations:** ^1^The National Institutes of Health, Eunice Kennedy Shriver National Institute of Child Health and Human Development, Bethesda, MD, United States; ^2^Mars Incorporated, Global Scientific Affairs, McLean, VA, United States; ^3^The WALTHAM Centre for Pet Nutrition, Leicestershire, United Kingdom

**Keywords:** human-animal interaction, animal-assisted interventions, anthrozoology, companion animals, service animals, child development, autism spectrum disorder, attention deficit hyperactivity disorder

Ten years ago, while reviewing the extant research literature on Human-Animal Interaction (HAI), a single question came to mind: “Why don't we know more about this topic?” (Griffin et al., 2011). A decade later the answer appears to have been, in part, a lack of infrastructure to organize and support stand-alone workshops and symposia at scientific conferences (and resulting edited volumes and journal articles) and concomitant sustained funding for rigorous research studies. As evidenced by this *Frontiers* Research Topic which includes 13 original data papers, the 10-year Public-Private Partnership (PPP) between the *Eunice Kennedy Shriver* National Institute of Child Health and Human Development (NICHD) and the WALTHAM® Centre for Pet Nutrition (WALTHAM®), a part of Mars Incorporated, has enabled the Human-Animal Interaction (HAI) field to make remarkable progress in, by scientific standards, a very brief timespan (McCune et al., 2020). In the final Opinion paper of this *Frontiers* Research Topic, Human-Animal Interaction Research: A Decade of Progress, we provide a commentary on how progress in basic and translational HAI research can be sustained as well as explore how ongoing challenges and untapped possibilities will impact the next decade of HAI research.

## Exemplars of Progress From this *Frontiers* Research Topic

The 13 papers that comprise the core of this Research Topic are a microcosm of the advances that have been made in HAI research, including the range of human and animal participants, the rigor of the study designs employed, and the diversity (and commonality) of measures used to capture mediator/moderator and outcome variables of interest.

### Human Participants

Although the initial studies funded under the PPP were constrained to those that would address the developmental mission of NICHD, the range of ages represented across the 13 studies is nonetheless impressive, with age of human participants ranging from 4-month-old infants (Hurley and Oakes, 2018) to adolescents (Pendry et al., 2018) and young adults (Syzmanski et al., 2018). The inclusion of a broad age range is important to document variations in HAI with development, and indeed, across the lifespan (Friedmann and Gee, 2018). Funding opportunities under the PPP were later expanded to include people with disabilities and those in need of rehabilitative services, further broadening the range of human participants (although this is not represented in this Research Topic because those studies are ongoing). In addition, the study participants represented in this Research Topic include children and adolescents from a wide variety of normative (e.g., Meints et al., 2018) and clinical populations (e.g., Gabriels et al., 2018; Schuck et al., 2018). Approximately half of the studies (7) represented are based on samples of typically developing children, with another including them as one of three subgroups. A range of clinical populations was represented in the studies, including individuals with Autism Spectrum Disorder (ASD, three studies), Attention Deficit Hyperactivity Disorder (ADHD, two studies), and incarcerated youth (one study). Despite the diversity of age and sample background characteristics, only one of the 13 studies (Jacobson and Chang, 2018) specifically examined the role of socioeconomic status (SES) as it relates to HAI and socioemotional outcomes. Racial/ethnic and cultural variables were not examined, and gender differences were not a focus of the analyses. One of the studies (MacLean and Hare, 2018) did not include humans at all; rather, it examined differences between assistance and detection dogs.

### Animal Participants

It is important to note that the studies employed or examined the relationship of children to a wide variety of animals, including dogs (the most common), horses (second), cats, and guinea pigs. While these studies undergo ethical review to ensure the welfare of the animals, the animal part of HAI is often not assessed, and the majority of the studies did not report the results of measures in the animals assessing stress at the behavioral or physiological level. Likewise, most did not report complete information on the age and gender of the animal, spaying/neutering status, and, if part of an intervention, what training and certification the animal had received and when. Additional information on the handlers of assistance or service dogs if they participated during an intervention would also aid replication and meta-analysis of these variables across a wide range of studies.

### Study Design

Four of the studies employed a randomized controlled trial design, and another employed an integrated control group design with each child being his or her own control (before and after a learning intervention and at the follow-up testing). One used survey data from a nationally representative study. The remaining studies used samples of convenience and employed surveys, observation, and laboratory testing to examine associations across a variety of types of HAI and child outcomes. Although progress has been made, more HAI studies are needed that employ randomized designs with sufficient sample sizes to ensure adequate power and control conditions that address competing explanations for detected effects.

### Measures and Methodology

The 13 studies in the Research Topic employed a wide range of measures, including salivary cortisol to assess Hypothalamic Pituitary Adrenal (HPA) axis reactivity and stress levels (Pendry et al., 2018; Pan et al., 2019), survey questions (Bures et al., 2019), and a genetic assay to look at the relationship between a single polymorphism in the oxytocin receptor and individual differences in response to HAI activities (Kertes et al., 2018).

Two studies specifically focused on examining the psychometric properties of HAI survey items related to children's attachment to their pets (Hart et al., 2018; Bures et al., 2019) and another used observational coding of children's interactions with animals in a naturalistic setting (Gabriels et al., 2018). Such methodology papers are critical to the advancement of HAI research.

## Looking to the Future

It is important to note that while the 13 research papers that make up this *Frontiers* Research Topic provide a fair representation of the types of research funded by the PPP, they are only a partial representation of such studies. Some of the PPP-supported investigations have already published their findings elsewhere, and others are still actively engaged in data collection and analysis. Likewise, important collections of HAI journal articles have been published on topics ranging from Animal-Assisted Interventions (AAIs) in special populations (McCune et al., 2017), the range of experiences children have with companion animals (Beetz et al., 2018), and the growing university-based infrastructure supporting HAI research (Serpell et al., 2017; O'Haire et al., 2018). Taken together, these efforts document the growth that has taken place over the last decade in HAI research. The overarching question now is what will be necessary to sustain such growth over the next 10 years?

The progress of the past decade covers a number of fronts: increased use of a single keyword to index publications (human-animal interaction), the widespread adoption of standardized terms and definitions (IAHAIO, 2014), increased methodological rigor in terms of study designs, standardized measurements (including genetic and biomarker assays), expansion of HAI research into new settings (Gee et al., 2017), and better communication across scientific disciplinary boundaries via scientific journals and professional societies (as evidenced by this *Frontiers* Research Topic). In order to build upon and sustain this growth there are a number of practices that need to be adopted or more widely utilized by the field, including but not limited to those outlined below.

### Use of Video

Although significant progress has been made in measuring human and animal behavioral and physiological responses, the “I” in HAI has largely gone unmeasured. The availability of low-cost high-definition video recording and editing makes it possible for researchers conducting HAI studies in laboratories, homes and other naturalistic settings to record HAIs as they take place for later coding and analysis. An exemplar of this approach (Guérin et al., 2018) demonstrates the power of having video from multiple studies with different populations to establish the psychometric properties of a behavior coding tool. Indeed, using video to record both training sessions prior to implementing interventions and the interventions themselves could make it possible to implement the intervention at another site with fidelity, allow for the coding of behaviors by raters blind to the intent of the intervention, and provide a resource for secondary data analyses and meta-analyses. A data library, Databrary, partially funded through grants from the NICHD and the National Science Foundation (NSF), has been established to promote the archiving and sharing of video data to improve the reproducibility of studies and accelerate the pace of scientific discoveries across a range of disciplines (Gilmore et al., 2018).

### Data Sharing

Clearly data archiving and sharing should not be limited to video data—HAI researchers should be encouraged to document and archive their datasets so that they can be used for secondary and meta-analytic studies by others in the field and by researchers from other disciplines who might code and analyze their data for variables of interest outside of HAI *per se* (e.g., language samples from young children interacting with their pets). One such data repository is the Data and Specimen Hub (DASH) funded by the NICHD, which provides archiving infrastructure for both electronic data and biospecimens (Hazra et al., 2018).

### Documenting and Manualizing Animal-Assisted Interventions

With the proliferation of AAIs comes the challenge of reproducibility and dissemination. Few AAI studies report their interventions in sufficient detail to allow for replication by another researcher or a practitioner in the field. Such information is critical to avoid failures of replication or improper implementation with clinical populations. The potential list of such details is extensive; e.g., how free is the animal to approach/withdraw or to interact with a subject? Is the subject allowed to touch the animal, and if so, in what way? How is the animal introduced to the subject? How is the intervention executed (such as reading to an animal?). These details would likely need to be included in supplementary material for a journal article or to be available for download on a lab website (e.g., Tufts Institute for Human-Animal Interaction, 2016). Likewise, there is a need to systematically report on various training and certification programs for therapy and service animals (e.g., what criteria were used to screen and select the therapy animals and their human owners or handlers for inclusion in the study? Were one or more certifications accepted for the animals/handlers?). It is important to document such details for future replication studies and potential adoption by other service providers for AAIs as well as visitation programs, especially those serving potentially vulnerable populations (e.g., individuals in hospitals and nursing homes).

### Inclusion

Although HAI research is carried out around the globe, the majority of studies conducted utilize convenience samples that are homogeneous in socioeconomic status, race/ethnicity, and cultural and religious background of the participants. Even fewer examine cross-cultural differences employing geographically diverse samples within and across countries (McCune et al., 2014). There is a great deal of work ahead for the field to fully explore these differences as they relate to HAI. These include the complex relationships between humans and other animals, with some seen as a source of food, others as beloved pets. As pet ownership becomes more accepted in some countries it is possible to treat this change as a natural experiment to examine how attitudes toward HAI change, and meet resistance, over time (Headey et al., 2008).

## Potential Pitfalls: What Will Not Contribute to Growth of HAI Research?

While the remarkable growth of HAI research over the last decade has added significantly to our scientific knowledge base, it is worth highlighting a few persistent and new trends that will not contribute to the accumulation of knowledge regarding HAI.

### Pets Do Not Confer Immortality

It is now commonplace to hear a news report about the health benefits of pet ownership, only to hear a similar story highlighting the exact opposite results (Herzog, 2011). To be clear, it is very important to examine the health benefits of companion animals, service animals, and AAIs generally, including the mechanisms by which those gains are realized (Serpell et al., 2017), and to acknowledge the strengths and limitations of the corpus of research used as the basis for statements by, for example, the American Heart Association (Levine et al., 2013) and the Mayo Clinic (Creagan et al., 2015).

Not all studies indicate benefits, and the data are not consistently positive or in agreement. At the heart of the contradictory findings is the fact that many studies on both sides of the health claims debate are generated by survey data that provide very limited information, often having a single item on pet ownership and a few health questions (Batty et al., 2017). Such studies cannot take into account reasons for (or to forego) pet ownership, the length of pet ownership, the relationship to the pet (positive and negative), including attachment, etc. (Ding et al., 2017). There are very likely both positive and negative effects of pet ownership on human health, with the aggregated data resulting in statistically significant associations depending on the sample and covariates employed in the analysis (Mueller et al., 2018). Obtaining a pet to obtain health benefits would be like seeking a spouse because married people tend to live longer (Tatangelo et al., 2017); one would likely be unhappy with the outcome.

A similar cautionary flag must also be raised regarding the benefits of service animals with select populations: although the preliminary research findings are promising (O'Haire and Rodriguez, 2018), many factors must be taken into account in the decision to obtain a service animal, including the ability to care for it (Crossman and Kazdin, 2015).

### Proliferation of Systematic Reviews and Meta-Analyses

There are times when the generation of new research findings in a given area warrant a similar rapid turn-around in the number of systematic reviews in that area of science. One such example is AAI studies with children who have Autism Spectrum Disorder (ASD), a topic which has generated a plethora of studies and two rapid cycle systematic reviews (O'Haire, 2013, 2016). That said, the number of systematic reviews and meta-analyses being conducted on this topic alone (Berry et al., 2013; Davis et al., 2015; Hill et al., 2018) indicates that disciplinary journals are being saturated with articles reviewing the same small set of papers with little or no cross-referencing of the other extant literature, including other systematic reviews. Journal editors and peer reviewers need to do at least a cursory search of the literature to see if manuscripts are sufficiently different from previous publications to warrant further consideration.

A similar issue will arise as data archiving becomes more common, allowing for secondary data analysis. Editors and reviewers must make sure any secondary papers are not reporting findings previously published by the original authors of the study. The bottom line is that to make meaningful contributions to the advancement of HAI research, investigators conducting systematic reviews and meta-analyses need to ensure that they are making a meaningful contribution to the current knowledge base and using the findings from their reviews and meta-analyses to make informed recommendations regarding future research directions, including topics, designs and measures. These reviews must not shy away from documenting null and negative effects, as well as noting adverse events that occur across similar interventions, as these are important for determining the risk level associated with an intervention and optimal ways to manage it.

## Conclusion: Build it and they Will Come

The PPP cannot claim credit for the significant advances in infrastructure supporting HAI research (university-based research centers, new dedicated journals, the second of two Frontiers Research Topics dedicated to HAI research), but it can claim some small role in encouraging their foundation and development (Esposito et al., 2011), with a concomitant rise in the adaptation of rigorous research designs and methodologies and the use of standardized measures which allow for comparison of methodologies and findings across studies. All of these factors support the accumulation of a growing empirical knowledge base and interdisciplinary research teams capable of conducting high quality HAI studies. It is hoped that the PPP has and will continue to contribute to the maturation of a field that provides timely evidenced-based information to guide topics ranging from pet ownership and social isolation to addressing the contributions of service animals to the social functioning of children with ASD and military service veterans with PTSD. The breadth of the HAI field is both a blessing and a curse, but those committed to advancing our understanding of the complex relationship between humans and animals would not have it any other way.

## Author's Note

The views expressed in this article are those of the authors and do not necessarily represent those of the National Institutes of Health, *Eunice Kennedy Shriver* National Institute of Child Health and Human Development, or the U.S. Department of Health and Human Services.

## Author Contributions

All authors listed have made substantial, direct and intellectual contributions to the work, and approved it for publication.

### Conflict of Interest

KH is employed by Mars Incorporated, Global Scientific Affairs, McLean, VA, United States. SM was employed by the Mars Incorporated, WALTHAM Centre for Pet Nutrition, Leicestershire, United Kingdom. The remaining author declares that the research was conducted in the absence of any commercial or financial relationships that could be construed as a potential conflict of interest.
